# Supplementary material for: Construction of the national fitness public service satisfaction model in China based on American Customer Satisfaction Index
Source: PLoS One. 2025 Jan 22;20(1):e0317551. doi: 10.1371/journal.pone.0317551 (PMC11753659; doi:10.1371/journal.pone.0317551)
Supplement: Attachment — Submitted filename: PONE-D-24-41589.docx [file pone.0317551.s002.docx]

**Construction of the national fitness public service satisfaction model in China based on American Customer Satisfaction Index**

**Originality**

1. Originality: Does the paper contain new and significant information adequate to justify

publication?

The Paper contain valuable information as considered significant for the justification and publication

The Paper contains valuable information as considered significant for the justification and publication.

**Relationship to Literature:**

This paper demonstrates a thorough understanding of the subject matter. Citations are also appropriate in a series of works. According to the review, substantial work is part of research.

**Methodology:**

The author supports the intellectual approach. The use of a survey method combined with an appropriate method result in a well-designed paper.

**Results**

During the detail review, it was discovered that each indicator of the subject under study is adequately presented. This reflected how each variable in the paper was interconnected. Under control variables analysis can be expanded.

**Implications for research, practice and/or society:**

The paper can be used to investigate problems and identify areas that are relevant to the subject. The conclusion and findings were based on the data provider’s applicability. Due to the use of long sentences, the sentence structure is unclear. This expression causes confusion for the average reader. However, the case is presented clearly due to the data and scientific approach used. The research technically implies field-specific terms.
