# Supplementary material for: Construction of the national fitness public service satisfaction model in China based on American Customer Satisfaction Index
Source: PLoS One. 2025 Jan 22;20(1):e0317551. doi: 10.1371/journal.pone.0317551 (PMC11753659; doi:10.1371/journal.pone.0317551)
Supplement: Attachment — Submitted filename: Response to reviewers.docx [file pone.0317551.s003.docx]

**Dear editor and reviewer:**

We extend our sincere gratitude for evaluating our research. According to your esteemed commentary, we have undertaken substantial revisions, highlighted in red within the revised manuscript. We are confident that these revisions have improved the quality and overall contribution of our research. We have crafted a comprehensive point-by-point response to address reviewer’s comments.

**The academic editor comments**

When submitting your revision, we need you to address these additional requirements.

1. Please ensure that your manuscript meets PLOS ONE’s style requirements, including those for file naming.
2. Please include your full ethics statement in the ‘Methods’ section of your manuscript file. In your statement, please include the full name of the IRB or ethics committee who approved or waived your study, as well as whether or not you obtained informed written or verbal consent. If consent was waived for your study, please include this information in your statement as well.

**Response**

1. We have modified the format of the paper to align with the style guidelines of PLOS ONE.
2. We have added an ethical statement to the methods section of the revised manuscript. See lines 229-230 of the revised manuscript.

**Reviewer 1 comments**

The paper can be used to investigate problems and identify areas that are relevant to the subject. The conclusion and findings were based on the data provider’s applicability. Due to the use of long sentences, the sentence structure is unclear. This expression causes confusion for the average reader. However, the case is presented clearly due to the data and scientific approach used. The research technically implies field-specific terms.

**Response**

In the revised manuscript, we have simplified and clarified any lengthy or unclear sentences.

**Reviewer 2 comments**

The study explores an interesting topic. However, there are significant issues should be addressed.
1. The manuscript primarily applies the ACSI model without offering significant modifications or extensions to suit the context of national fitness services in China. The lack of theoretical innovation limits the paper's contribution to academic literature.

**Response**

As the reviewer noted, this paper constructs a satisfaction model for China’s national fitness services based on the ACSI framework, which may lack significant theoretical innovation. In China, despite the national fitness being a strategic priority, there is still a notable absence of scientific models to assess satisfaction with these services. The ACSI model has gained increasing recognition and application in evaluating the satisfaction with non-sports public services in China. Therefore, the satisfaction model developed in this study has practical significance. It can guide the reform of the supply side of national fitness, and serve as a foundation for further in-depth research by scholars in China.

2.The sampling strategy is inadequately detailed. While 1133 valid responses were collected, the demographic distribution (e.g., age, gender, education) is imbalanced, which could bias the results.

**Response**

Firstly, we have included a more detailed sampling strategy in the revised manuscript. See lines 232-242 of the revised manuscript. The specific modifications are outlined as follows:

Using a convenience sampling method, the study delivered online questionnaires and paper questionnaires. The digital questionnaire was distributed via the Questionnaire Star platform (https://www.wjx.cn), and shared in fitness-related WeChat groups. In addition, effective epidemic control of the epidemic in China at the time allowed residents in unaffected areas to resume outdoor activities, following regular nucleic acid tests. This enabled the research team to visit various district national fitness centers across Guangzhou to distribute paper questionnaires. The team successfully coordinated with center managers and staff to rally local residents who were engaged in fitness activities. With residents’ consent, paper questionnaires were distributed and collected on-site. The study distributed 100 questionnaires in each of Huadu, Baiyun, and Panyu districts; 200 in each of Liwan and Haizhu districts; 240 in Tianhe district; and 80 in Yuexiu district.

Secondly, convenience sampling using in the study may have resulted in an imbalance in the demographic distribution of the sample. This limitation has been acknowledged in the revised manuscript. See lines 374-380 of the revised manuscript. The specific modifications are outlined as follows:

Using convenience sampling method may bias the sample towards easily accessible populations. For example, online questionnaires might attract individuals who frequently use the internet, while paper questionnaires might draw more individuals who often engage in outdoor activities. The representativeness of the sample and are somewhat constrained. As illustrated in Table 2, there is a low percentage of respondents in the age groups 21-30, 51-60, and over 60 years old. Future studies should control the potential biases introduced by convenience sampling.

3. The use of a hybrid data collection method (online and paper-based) introduces potential inconsistencies in response quality, which are not addressed in the discussion.

**Response**

In the “Limitations and Future Research” section of the revised manuscript, we have provided an in-depth discussion on the potential inconsistencies in response quality that could arise from employing a hybrid method. See lines 387-397 of the revised manuscript. The specific modifications are outlined as follows:

To response the challenges posed by the degradation of survey environments and rising execution costs, the use of hybrid surveys—combining multiple data collection methods within a single project—has become common practice in the United States and Europe. Research indicates that the mode of information delivery and the presence of interviewers can influence respondents’ answering behaviors. In this study, the paper questionnaire yielded a higher effective response rate compared to the digital version, likely because respondents were more diligent when completing a physical questionnaire under the researcher supervision. In addition, national fitness centers in China are predominantly government-operated, the involvement of fitness center managers may have led participants to perceive the survey as more official. Nonetheless, this study applied a uniform criterion for discarding invalid questionnaires, ensuring consistency and quality across both online and paper-based surveys.

4. The study’s focus on Guangzhou, China, restricts its applicability to other regions or countries. The findings, while useful for local policymakers, lack broader relevance unless comparisons or implications for other regions are discussed.

**Response**

Owing to the varying in epidemic control measures across different regions in China, this study focused its investigation on Guangzhou, which limits the generalizability of its findings to some extent. This limitation is discussed in the “Limitations and Future Studies” section of the revised manuscript. See lines 381-386 of the revised manuscript. The specific modifications are outlined as follows:

China’s vast geographical expanse and economic disparities lead to variations in the level of public services, which in turn influence residents’ perceptions of national fitness services. Owing to the varying in epidemic control measures across different regions in China, this study focused its investigation on Guangzhou, the generalizability of the findings is limited to some extent. To enhance the applicability of the results, future research should consider conducting a nationwide survey to enable broader validation of the model presented here.

5. While the results demonstrate significant pathways, certain rejected hypotheses (e.g., public expectation directly influencing public satisfaction) are not adequately explained. The discussion could delve deeper into why these relationships failed to materialize and what that implies for theory and practice.

**Response**

In the discussion section of the revised manuscript, the two rejected assumptions (public expectation → public satisfaction; public complaints → public trust) were discussed in detail. See lines 320-321, 327-331 and 365-372 of the revised manuscript. The specific modifications are outlined as follows:

Public expectation exerts no direct, statistically significant influence on public satisfaction (path coefficient of 0.039, p>0.05), it significantly impacts perceived quality (path coefficient of 0.445, p<0.01), which in turn significantly impacts public satisfaction. Public expectation is commonly understood as the anticipated level of service, product, or policy performance, serving as a comparative benchmark. In service quality assessment, satisfaction is affected on the expectation-perception gap, namely the discrepancy between the actual service quality experienced and the expectations previously set. Satisfaction is achieved when the perceived performance meets or exceeds these expectations (Zhang et al.,2022). This highlights the importance of perceived quality in shaping public satisfaction.

The study found that public complaints have no direct effect on public trust (path coefficient = -0.008, P>0.05). This could be because the complaints examined in this study were primarily behavioral outcomes related to specific events or experiences. Whereas trust encompasses a broader evaluation of reliability, integrity, and consistent performance over time. As a result, complaints about specific events may not significantly influence the overall public trust in government services. In summary, high levels of public satisfaction reduce complaints about public sports services and foster trust in the government’s national fitness initiatives.

6. The practical recommendations are too general. For example, suggesting improvements in perceived quality and perceived value does not provide actionable strategies for policymakers or service providers.

**Response**
In the conclusion section of the revised manuscript, we have outlined the practical recommendations in detail. See lines 405-411 of the revised manuscript. The specific modifications are outlined as follows:

The most important practical implication of this study is that government departments should prioritize improving the quality of national fitness public services, such as enhancing the accessibility of fitness venues, upgrading the professional competence of fitness instructors, expanding efforts to offer free or low-cost access to venues, and strengthening channels for complaints and oversight. Enhancing service quality will enable national fitness services to better align with public expectations, boost the perceived value and satisfaction, reduce complaints, and ultimately build greater public trust.

**Reviewer 3 comment**

There is a minor issue with the manuscript such as language (line 47: replace "is" with "was").

**Response**

We have fixed this language error. See line 49 of the revised manuscript.

**Reviewer 4 comments**

**Abstract:**
• Consider enhancing the abstract by including specific details for better clarity and impact:
1. Briefly provide the rationale for constructing the national fitness public service satisfaction model to help readers quickly understand its significance and relevance.
2. Incorporate concrete values and statistical significance (e.g., path coefficients, p-values) for key results, enabling readers to grasp the quantitative aspects of the findings at a glance.
3. Ensure the conclusion succinctly summarizes the core findings while offering specific and actionable suggestions based on the results.

**Response**

In the abstract section of the revised manuscript, we have briefly provided the rationale for constructing the national fitness public service satisfaction model, included concrete values and statistical significance in the results, and revised the conclusion to summarize the study’s core findings. Additionally, we have structured the abstract into four distinct sections: objective, methods, results, and conclusion. See lines 45-70 of the revised manuscript.

**Introduction:**
1. Consider revising the stated objectives and hypotheses to align with the study's main findings, particularly the reliability and validity of the measurement model.
2. It is recommended to clearly articulate the rationale for using the ACSI model in this study, further reinforcing the research’s background and motivation.

**Response**

In the introduction of the revised manuscript, we have refined the objectives and hypotheses, and clearly articulated the rationale for using the ACSI model. See lines 126-134 of the manuscript. The details of the modifications are outlined as follows:

Moreover, there is a deficiency in the availability of a scientific model to accurately assess satisfaction with national fitness services. The ACSI model, recognized for its maturity, has been widely adopted by public sectors worldwide as a reliable tool for assessing public satisfaction. In China, an increasing number of public entities have introduced this model. Therefore, the objective of this study was to develop the dimensions and corresponding indexes for a national fitness service model tailored to China based on the ACSI framework, and further to validate the reliability and validity of the measurement model and the structural model’s fit level using survey data. Ultimately, the study elucidated the complex interrelationships among the various dimensions of the model.

**Methodology:**
1. The Methods section states that a total of 1,494 questionnaires were collected, comprising both digital (x%) and paper-based (x%) responses. Of these, 1,133 were ultimately included as valid responses. To enhance the transparency and rigor of the methodology, please provide a breakdown of the valid questionnaires by type (e.g., percentage or number of valid digital versus paper-based responses).
2. Additionally, one of the exclusion criteria mentioned is a completion time of less than five minutes for digital questionnaires. It would be beneficial to include a rationale for this specific threshold to justify its appropriateness in assessing response quality. Furthermore, please clarify whether similar criteria were applied to paper-based questionnaires, and if so, how completion time was assessed to ensure consistency across both formats.
3. Consider revising or expanding the statistical analysis to provide a more comprehensive overview of the techniques employed in this section.

**Response**

In the revised manuscript, we have included a breakdown of the valid questionnaires by type, and clarified the criteria for deeming a digital questionnaire invalid if it is completed in less than 5 minutes, a threshold that also applies to paper questionnaires. Furthermore, we have expanded the overview of the statistical analysis techniques employed in this section. See lines 247-264 of the revised manuscript. The specific modifications are outlined as follows:

A preliminary survey was conducted with 15 postgraduate students specializing in social and leisure sport, established an average completion time of approximately 5 minutes. For online questionnaires, the system automatically tracked the time completing each form. For paper questionnaires, they were distributed in a centralized manner, research team members monitored completion times on-site, submitted in under 5 minutes were deemed invalid. Finally, 1133 valid responses were identified, resulting in an effective response rate is 75.8%. Of the valid response, 438 were from digital questionnaire, 695 were from paper questionnaires.

The study used SPSS 22.0 software to analyze the demographic characteristic of the survey participants, as detailed in Table 2. To assess the reliability and validity of the measurement models, SPSS 22.0 and AMOS 16.0 software were used to calculate key metrics, including Factor Loadings, Average Variance Extracted (AVE), Composite Reliability (CR), and Cronbach’s alpha coefficient. Additionally, AMOS 16.0 software was used to evaluate the fit level of the structural model by calculating various fit indices, including the chi-square to degrees of freedom (χ²/df) ratio, Goodness of Fit Index (GFI), Adjusted Goodness of Fit Index (AGFI), Incremental Fit Index (IFI), Tucker-Lewis Index (TLI), Comparative Fit Index (CFI), Normed Fit Index (NFI), Relative Fit Index (RFI), Parsimonious Normed Fit Index (PNFI), and Parsimonious Goodness of Fit Index (PGFI).

**Results:**
1. For clarity, consider adding the meaning of any abbreviations used in the tables directly within the table captions or footnotes.

**Response**

We have added the meaning of abbreviations in Table 5. See lines 312-313 of the manuscript.

**Discussion:**
1. Consider discussing the reliability and validity results more comprehensively. For instance, address any observed lower values in factor loadings or AVE and their potential implications.

**Response**

We apologize for any confusion caused by the unclear language in the original manuscript. In revised manuscript, we have clarified that the factor loadings and average variance extracted (AVE) for all items in our study surpass the recommended threshold. Please refer to lines 282-288 of the revised manuscript. The specific explanation is as follows：

A factor loading exceeding 0.71 is considered excellent, above 0.63 is very good (Cyomery, 1973). Table 3 reveals that the all items’ factor loadings exceed the threshold of 0.63. A higher AVE value indicates stronger convergence validity, a threshold of 0.5 for AVE is commonly considered acceptable (Wu, 2017). AVE values for the dimensions of public expectation, perceived quality, perceived value, public satisfaction, public complaint, and public trust all surpass 0.5.

2. Lines 290–305: The authors are encouraged to strengthen this section by incorporating references to related studies or theories rather than relying primarily on examples. This approach will provide a more robust theoretical framework and better situate the findings within the context of existing literature.

**Response**

We have included pertinent literature to provide context and depth to our discussion of the results. See lines 337-343 of the revised manuscript. The specific modifications are outlined as follows:

Perceived value is essentially how customers perceive a product or service, in which customers evaluate costs versus benefits (Yum,2024). High-quality services or products—characterized by attributes such as reliability, effectiveness, and superiority—tend to lower perceived costs relative to the benefits received. Therefore, the higher the quality of a service, the greater the perceived value, and increasing the likelihood of public satisfaction. This positive correlation among quality, perceived value, and satisfaction has been substantiated by numerous studies, such as Yu et al. (2014), Hu et al. (2009), Kuo et al. (2009).

3. Please consider acknowledging the limitations of this study that may link to the future study.

Response

In the revised manuscript, we have added a “Limitations and furure study” section within the discussion. See lines 374-397 of the revised manuscript. The specific modifications are outlined as follows:

This study has several limitations. Firstly, using convenience sampling method may bias the sample towards easily accessible populations. For example, online questionnaires might attract individuals who frequently use the internet, while paper questionnaires might draw more individuals who often engage in outdoor activities. The representativeness of the sample and are somewhat constrained. As illustrated in Table 2, there is a low percentage of respondents in the age groups 21-30, 51-60, and over 60 years old. Future studies should control the potential biases introduced by convenience sampling.

Secondly, China’s vast geographical expanse and economic disparities lead to variations in the level of public services, which in turn influence residents’ perceptions of national fitness services. Owing to the varying in epidemic control measures across different regions in China, this study focused its investigation on Guangzhou, the generalizability of the findings is limited to some extent. To enhance the applicability of the results, future research should consider conducting a nationwide survey to enable broader validation of the model presented here.

Thirdly, to response the challenges posed by the degradation of survey environments and rising execution costs, the use of hybrid surveys—combining multiple data collection methods within a single project—has become common practice in the United States and Europe. Research indicates that the mode of information delivery and the presence of interviewers can influence respondents’ answering behaviors. In this study, the paper questionnaire yielded a higher effective response rate compared to the digital version, likely because respondents were more diligent when completing a physical questionnaire under the researcher supervision. In addition, national fitness centers in China are predominantly government-operated, the involvement of fitness center managers may have led participants to perceive the survey as more official. Nonetheless, this study applied a uniform criterion for discarding invalid questionnaires, ensuring consistency and quality across both online and paper-based surveys.

**Conclusion:**
• The conclusion should provide a concise summary of the study’s key findings while offering clear suggestions and specific directions for future research. Avoid repeating details from the Methods and Results sections to maintain a focused and impactful conclusion.

**Response**

We have refined our conclusions to capture our key findings and to propose actionable insights with practical implications. See lines 401-411 of the revised manuscript. The specific modifications are outlined as follows:

Among these variables, public expectation directly affects perceived quality, which in turn directly affects perceived value and public satisfaction. Public satisfaction then directly impacts public complaints and public trust. In the model, perceived quality plays a central role. The most important practical implication of this study is that government departments should prioritize improving the quality of national fitness public services, such as enhancing the accessibility of fitness venues, upgrading the professional competence of fitness instructors, expanding efforts to offer free or low-cost access to venues, and strengthening channels for complaints and oversight. Enhancing national fitness service quality will enable services to better align with public expectations, boost the perceived value and satisfaction, reduce complaints, and ultimately build greater public trust.
